# Laparoscopic training workshop to assess medical students’ skill acquisition and interest in surgical careers

**DOI:** 10.1186/s12909-024-05708-4

**Published:** 2024-07-03

**Authors:** Pin-Chun Chen, Po-Wen Yang, Yi-Kai Kao, Chia-Hung Chen, Chih-Jong Tsai, Yi-Chieh Chen, Ling-Chiao Song, Kai Lung Tsai, Richard C. Wu, Chih-I Chen

**Affiliations:** 1 Division of Colon and Rectal Surgery, Department of Surgery, E-DA Hospital, I-Shou University, Kaohsiung, Taiwan; 2 Division of Colon and Rectal Surgery, Department of Surgery, E-DA Cancer Hospital, I-Shou University, Kaohsiung, Taiwan; 3 Executive Master of Business Administration, National Sun Yat-sen University, Kaohsiung, Taiwan; 4 Division of General Surgery Medicine, Department of Surgery, E-Da Hospital, I-Shou University, Kaohsiung, Taiwan; 5 School of Medicine, I-Shou University, Kaohsiung, Taiwan; 6 Division of Colon and Rectal Surgery, Department of Surgery, Kaohsiung Chang Gung Memorial Hospital, Kaohsiung, Taiwan; 7 Department of Urology, E-Da Hospital, Kaohsiung, Taiwan; 8 Department of Information Engineering, I-Shou University, Kaohsiung, Taiwan; 9 Department of Nursing, I-Shou University, Kaohsiung, Taiwan

**Keywords:** Laparoscopic surgery, Surgical training, Medical education, Training workshop

## Abstract

**Background:**

With its minimally invasive approach, laparoscopic surgery has transformed the medical landscape. As the demand for these procedures escalates, there is a pressing need for adept surgeons trained in laparoscopic techniques. However, current training often falls short of catering to medical school education. This study evaluates the impact of a custom-designed laparoscopic training workshop on medical students’ surgical skills and career aspirations.

**Methods:**

This prospective experimental study was conducted at the E-Da hospital in Kaohsiung City, Taiwan. Medical students from Taiwanese medical schools undergoing Clerk 5, Clerk 6, and Postgraduate Year 1 and 2 were invited to participate. Medical students (*n* = 44) underwent an endoscopic skill training workshop consisting of lectures, box training, and live tissue training. The trainees performed multiple tasks before and after training using our objective evaluation system. The primary outcome was assessed before and after training through a questionnaire assessing the influence of training on students’ interest in surgery as a career. The secondary outcome measured improvement in skill acquisition, comparing the task completion time pre- and post-workshop. For the primary outcome, descriptive statistics were used to summarize the questionnaire responses, and paired t-tests were performed to determine significant changes in interest levels post-workshop. For the secondary outcome, paired t-tests were used to compare the time recorded pre- and post-training.

**Results:**

Post-training, participants exhibited significant proficiency gains, with task completion times reducing notably: 97 s (*p* = 0.0015) for Precision Beads Placement, 88.5 s (*p* < 0.0001) for Beads Transfer Exercise, 95 s (*p* < 0.0001) for Precision Balloon Cutting, and 137.8 s (*p* < 0.0001) for Intracorporeal Suture. The primary outcome showcased an increased mean score from 8.15 pre-workshop to 9.3 post-workshop, indicating a bolstered interest in surgery as a career. Additionally, post-training sentiment analysis underscored a predominant inclination toward surgery among 88% of participants.

**Conclusion:**

The custom-designed laparoscopic workshop significantly improved technical skills and positively influenced students’ career aspirations toward surgery. Such hands-on training workshops can play a crucial role in medical education, bridging the gap between theoretical knowledge and practical skills and potentially shaping the future of budding medical professionals.

## Introduction

Laparoscopic surgery has revolutionized the medical field owing to its minimally invasive nature and relatively rapid patient recovery [1]. As the global demand for these procedures rises, an increasing need for competent surgeons skilled in laparoscopic techniques has arisen [2, 3]. However, current laparoscopic training workshops often align poorly with the needs of medical school education. The Fundamentals of Laparoscopic Surgery Program, organized by the Society of American Gastrointestinal and Endoscopic Surgeons, is the leading laparoscopic training in the United States [4]. While such intensive training is crucial for residents who will begin operating on real patients, it may be excessive for medical students seeking a broad and meaningful introduction to diverse experiences.

Existing research has previously shown the profound impact of hands-on experience on shaping medical students’ perspectives. Earlier exposure to hands-on training is linked to the readiness of medical students and deciding if this surgical specialty aligns with their professional aspirations [5, 6]. Several studies have previously emphasized the value of early hands-on experience in medical education. One prior study [7] demonstrated that early hands-on experience enhances the surgical clerkship experience and improves medical students’ perceptions of surgery as a career. Likewise, a study by Al-Heeti [8] illustrated similar results.

Hands-on laparoscopic experience has been shown to considerably boost students’ technical skills, enhance learning, maintain motivation, and increase interest in surgery as a future career [9, 10]. However, medical students’ exposure to laparoscopy is predominantly confined to watching live camera streams during surgeries [11]. Opportunities to hone their laparoscopic skills are often constrained because of limited hands-on experiences in their current curricula [12, 13]. Medical institutions should ideally equip students with technical abilities that mirror real surgical practices, including hands-on experience with laparoscopic techniques. Medical training deeply values hands-on learning [5]. Ideally, students should be offered substantial opportunities for active involvement [2].

Our study seeks to bridge this aforementioned gap by examining whether hands-on laparoscopic experience can shape medical students’ predilection for surgery as a career choice. Additionally, we aim to further evaluate the efficacy of our workshop format in imparting these essential skills.

## Methods

### Study design and participants

We conducted a prospective experimental study to assess the effectiveness of our custom-designed laparoscopic training workshop. The study recruited 44 medical students from medical schools in Taiwan. Medical students from Clerk 5, Clerk 6, Postgraduate Year 1 (PGY) 1, and PGY 2 were invited to participate. When assessing the laparoscopic experience of our participants, we found a range of familiarity and hands-on skills. Students with no prior exposure to any laparoscopic techniques or procedures were classified as “Novices,” while those with experience limited to observational roles in laparoscopic procedures were classified into the “Observer” category. Some participants were “Simulator-Trained,” having practiced with laparoscopic simulators, while a final group was “Clinically Experienced,” showcasing hands-on expertise from actual laparoscopic patient procedures.

### Intervention

**Fig. 1:**
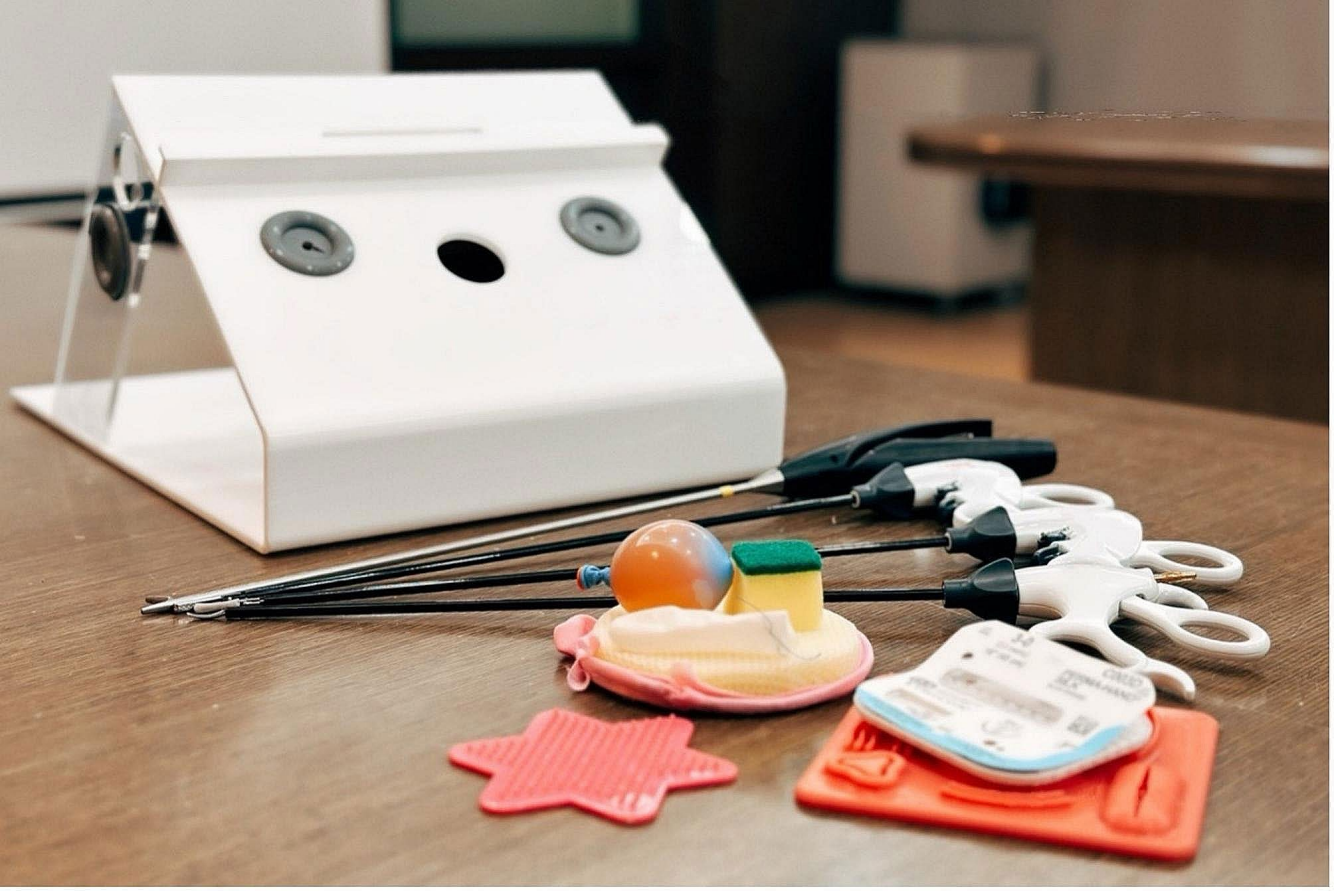
Training box. Training box containing laparoscopic instruments (graspers, scissors, and needle holders), a camera system, and materials for practicing skills (beads, balloons, and sutures)

All participants underwent our laparoscopic training workshop, structured to provide a comprehensive introduction to the fundamental laparoscopic principles and techniques. The workshops comprised lectures, box training (Fig. 1), and live tissue training. The curriculum was standardized to ensure each student received consistent training.

**Fig. 2:**
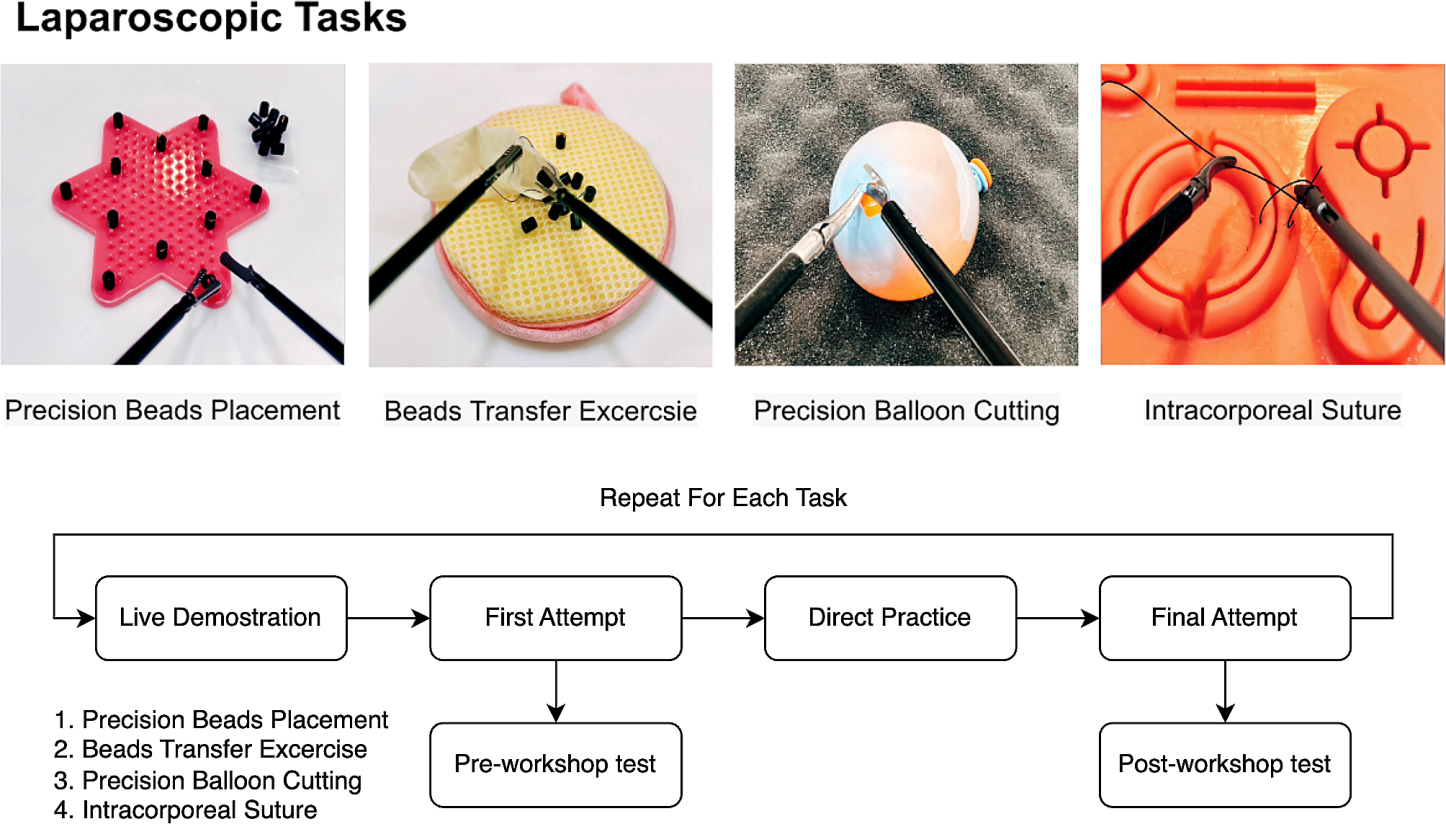
Laparoscopic tasks. **(A)** Precision bead placement, requiring precise positioning of beads using graspers; **(B)** Beads transfer exercise, focusing on instrument coordination and handling; **(C)** Precision balloon cutting, involving delicate peeling of an outer balloon without puncturing the inner water-filled balloon; **(D)** Intracorporeal suturing, necessitating mastery of suturing techniques within a confined space

Our laparoscopic training module comprised four distinct tasks (Fig. 2), each meticulously designed to hone specific skill sets:

Precision Bead Placement: Participants were required to use laparoscopic graspers to meticulously position tiny beads in specified locations, emphasizing precision and dexterity.

Beads Transfer Exercise: This task involved the transfer of beads from one location to another, focusing on coordination and handling of the laparoscopic instruments.

Precision Balloon Cutting: Participants were presented with a setup where a water-filled balloon was enclosed within an outer deflated balloon. The aim of the task was to delicately peel the outer balloon using laparoscopic instruments without puncturing the inner water-filled balloon, underscoring precision and control.

Intracorporeal Suturing: This advanced task necessitated participants to master the technique of suturing within a confined space, mirroring actual surgical conditions and emphasizing technical proficiency and spatial awareness.

Sessions were split into four sections, one for each laparoscopic task. Each followed a similar format (Fig. 2). Sessions began with a live task demonstration. During the demonstration, students were permitted to ask questions and request that portions of the task be repeated. Following the demonstration, students completed their first attempt at the task without assistance, and their times were recorded. After completing their first attempt, all students were allowed approximately 1 h of directed practice time. The instructor answered questions, provided feedback, and performed additional demonstrations throughout the session. When only 5 min remained in the section, students were instructed to complete a final attempt at the task. The final attempt was unassisted and recorded for timing. Students were given a 5-min break before moving on to the next task.

In addition to the laparoscopic tasks performed using the training box, the workshop also included a live tissue training component using pig intestines. This hands-on practice aimed to provide students with an introductory exposure to advanced techniques, such as the use of endoGIA staplers and intestinal anastomosis. However, as these practices were considered too complex for the skill level of the participants, the live tissue training was not included in the study protocol or assessed as part of the outcomes.

### Primary outcome measure

To ascertain the potential influence of our training workshop on the students’ career aspirations, participants were asked to complete a 12-item questionnaire. This survey aimed to assess the extent to which the laparoscopic training influenced their interest in pursuing surgery as a future career. The questionnaire was given to the participants post-training, and a self-rated scale was utilized for the pre- and post-test assessments. This scale ranged from 0 (indicating no interest in pursuing surgery) to 10 (indicating a strong desire to pursue surgery as a career).

Pre-test: Participants were asked, “How would you rate your interest in choosing surgery as a future career?” on a 0–10 scale.

Post-test: After completing the laparoscopic training workshops, participants were asked, “Following this workshop, how would you now rate your interest in choosing surgery as a career?” with answers ranked on the same scale.

### Secondary outcome measure

To evaluate improvements in skill acquisition, the time taken to complete a standardized laparoscopic task was recorded for each student before and after the training workshop, with task-specific penalties in the form of added time being assessed when applicable (cutting the balloon with water leakage, insecure knot, etc.). The result was the change in the time taken to complete this task post-training compared with their initial attempt.

### Statistical analysis

For our primary outcome, descriptive statistics were applied to summarize questionnaire responses, and paired t-tests were performed to determine any significant changes in interest levels post-workshop. For our secondary outcome, paired t-tests were utilized to compare the times recorded before and after the training. A *p*-value of less than 0.05 was considered statistically significant.

### Ethical considerations

Prior to the initiation of the study, ethical approval was obtained from the Institutional Review Board of the E-Da hospital, R.O.C (No. 2,023,018). All participants provided written informed consent, ensuring they were aware of the study’s purpose, procedures, potential risks, and benefits.

## Results

The study included 44 participants (Table 1) from different academic backgrounds within the medical curriculum. Of these, 21 participants (48%) were women, and the remaining 23 (52%) were men. Regarding their academic status, 9 participants (20%) were from Clerk 5, 19 (42.2%) from Clerk 6, 16 (26.6%) from PGY 1, and the last 5 participants (11.1%) were from PGY 2.

**Table 1:** Baseline characteristics of participants

No. of Participants	44
**Clinical Years**	
Clerk 5	9 (20%)
Clerk 6	19 (42.2%)
PGY 1	16 (26.6%)
PGY 2	5 (11.1%)
**Sex**	
Men	23 (52%)
Women	21 (48%)
**Laparoscopic Experience of Participants**	
Novice	4 (8.8%)
Observer	22 (48.8%)
Simulator-Trained	14 (31.1%)
Clinically Experienced	5 (11.1%)

PGY = postgraduate year

Within our participant pool, there was a varied distribution of laparoscopic experience. A minority (4 participants, 8.8%) were classified as “Novice” with no prior exposure to laparoscopic techniques. The majority were “Observers,” constituting 22 participants (48.8%), of which 14 participants (31.1%) had gained experience through laparoscopic simulators, labeled as “Simulator-Trained.” Finally, 5 participants (11.1%) had direct patient procedural exposure, falling under the “Clinically Experienced” category.

**Fig. 3:**
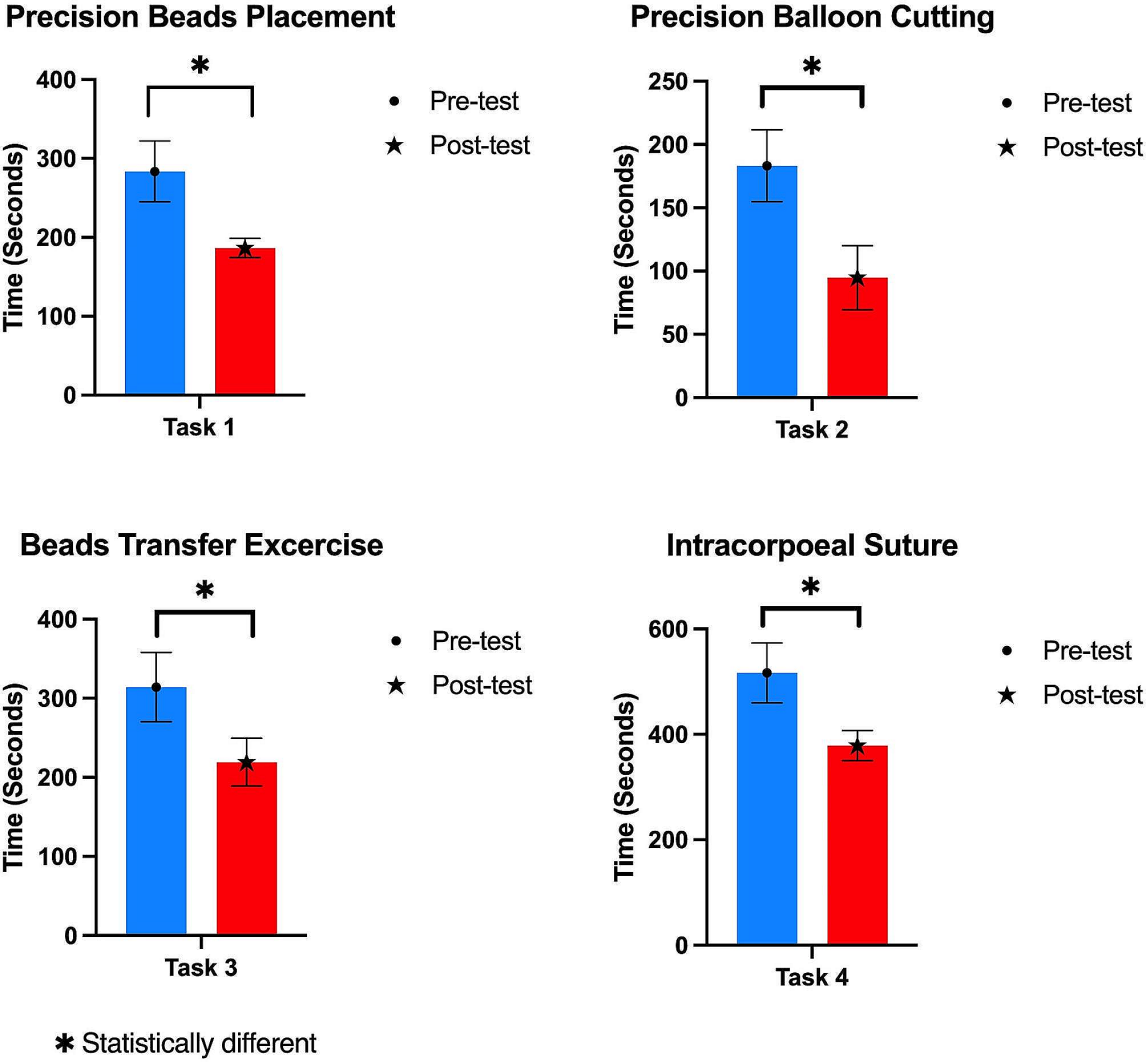
Comparison of pre-test and post-test completion times for various tasks

For all tasks, post-test completion times showed a significant reduction compared with pre-test times (Fig. 3).

Task 1 was Precision Bead Placement. The average completion time decreased from 283.6 s (SD ± 38.6) during the pre-test to 186.6 s (SD ± 12.2) during the post-test. The average decrease of 97 s was significant (*p* = 0.0015). Task 2 was the Beads Transfer Exercise. The completion time decreased from an average of 183.3 s (SD ± 28.4) during the pre-test to 94.8 s (SD ± 25.4) during the post-test. The average decrease of 88.5 s was significant (*p* < 0.0001). Task 3 was Precision Balloon Cutting. The completion time decreased from an average of 314.3 s (SD ± 43.8) during the pre-test to 219.3 s (SD ± 30.3) during the post-test. The average decrease of 95 s was significant (*p* < 0.0001). Task 4 was Intracorporeal Suturing. The completion time decreased from an average of 516.4 s (SD ± 56.7) during the pre-test to 378.6 s (SD ± 28.6) during the post-test. The average decrease of 137.8 s was significant (*p* < 0.0001).

The survey response rate was 97% (43/44). In investigating the impact of laparoscopic workshops on students’ inclination toward choosing surgery as a prospective career, a quantitative assessment was conducted before and after the workshop intervention. The data was analyzed using descriptive statistics to determine the central tendencies and dispersion.

**Fig. 4:**
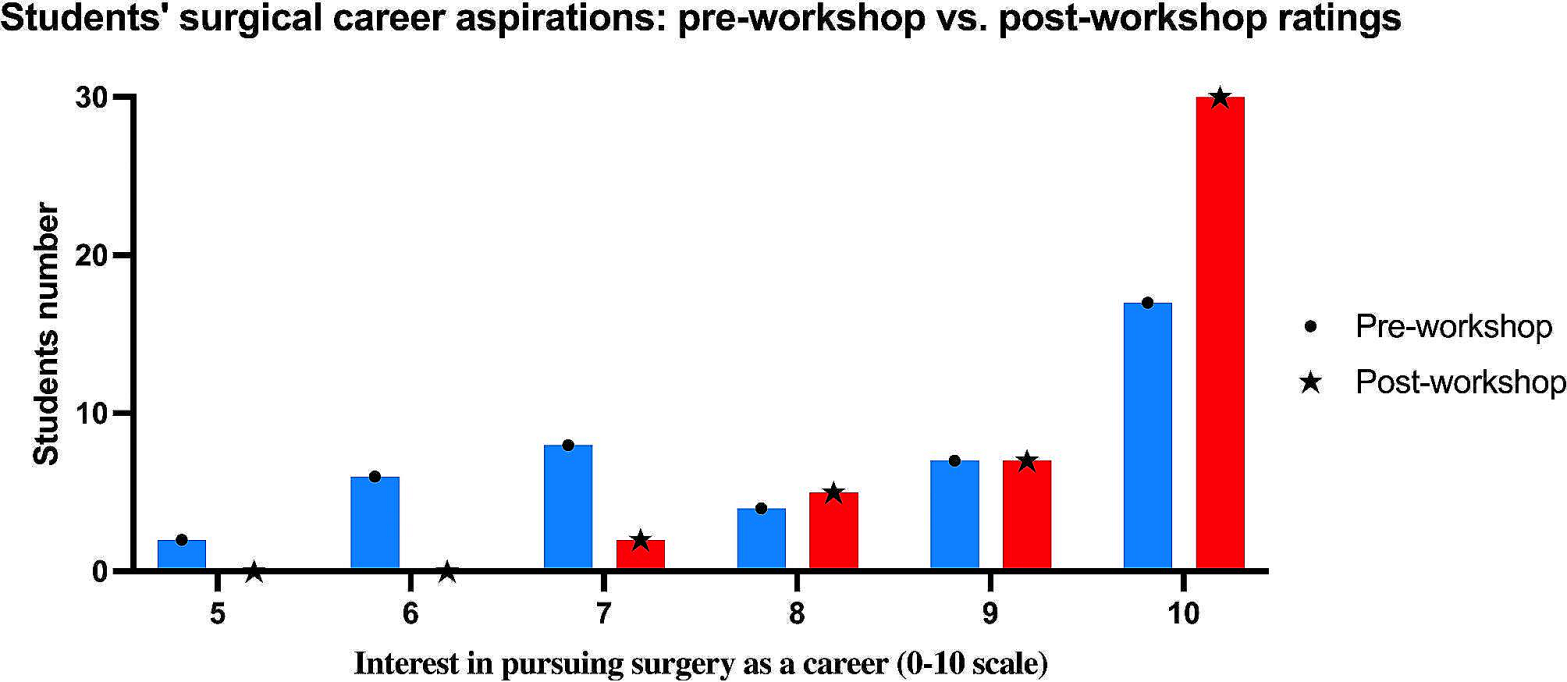
Students’ surgical career aspirations: pre-workshop vs. post-workshop ratings

The pre-workshop evaluation revealed a mean score of 8.15, with a median of 8. The scores ranged from a minimum of 5 to a maximum of 10, indicating a moderate to high initial interest among the participants in pursuing surgery as a career. Conversely, following the completion of the laparoscopic training workshops, there was a discernible shift in the participants’ responses. The post-workshop mean score escalated to 9.3, and the median increased to 10, the maximum value of the scale. The narrowed range of scores, from 7 to 10, underscores a heightened and more uniform interest in surgery as a career choice among the participants (Fig. 4).

**Fig. 5:**
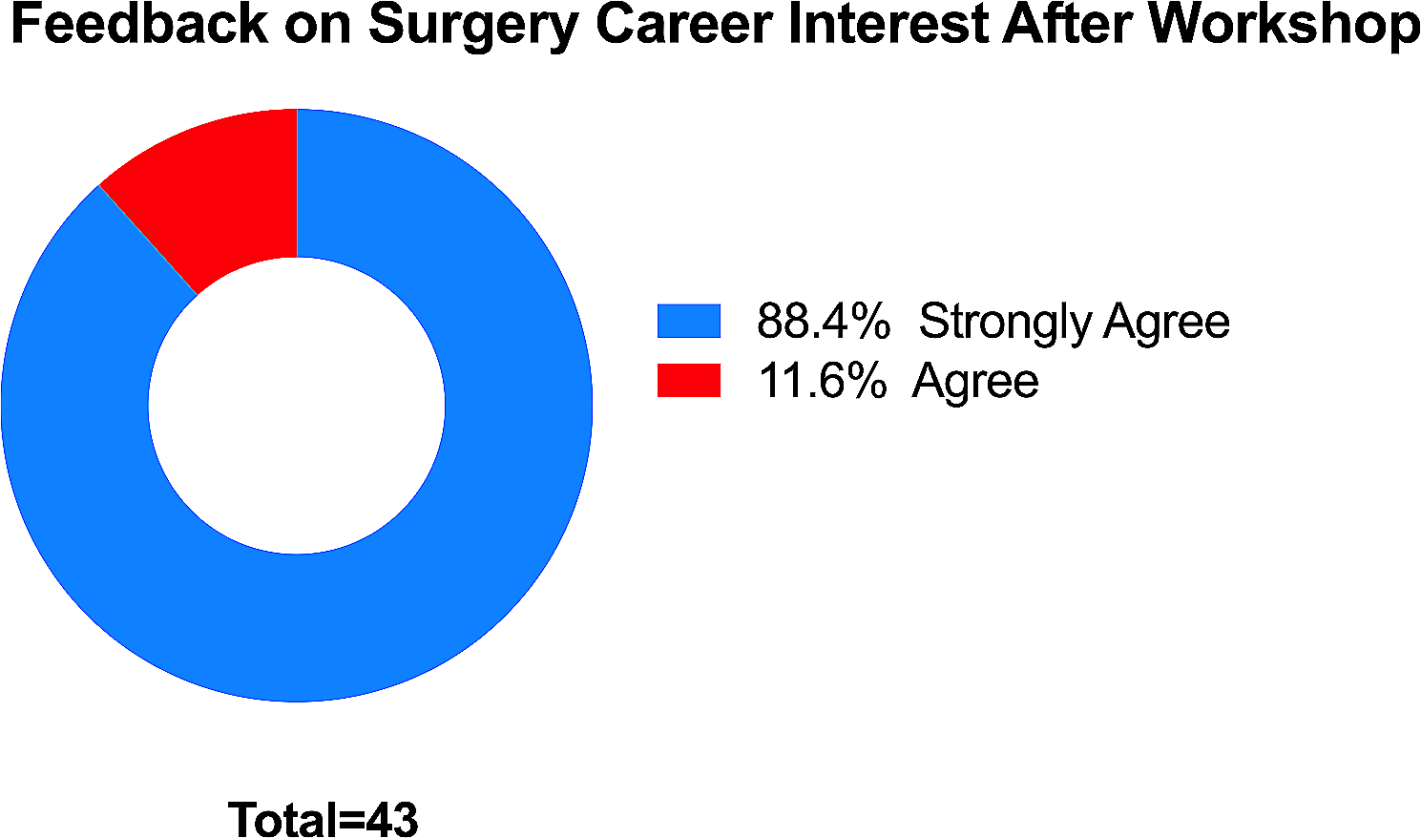
Feedback on surgery career interest after the workshop

Moreover, utilizing a five-point Likert scale post-hands-on training with a laparoscopic training box, we further probed into the post-training sentiments of the students. Most participants (38/43) chose the “strongly agree” option when questioned about their interest in surgery as a profession, further underscoring the positive influence of the hands-on experience. Only a minority of 6 participants merely agreed, without any participant choosing the “neutral,” “disagree,” or “strongly disagree” options (Fig. 5).

In Taiwan, PGY1 students can choose general training or specialized training focusing on a specific discipline such as internal medicine, surgery, gynecology, or pediatrics. In our study, 16 participants (26.6%) were PGY1 students at the time of the laparoscopic training workshop. We conducted a follow-up to determine how many of these PGY1 students subsequently chose to join the surgery group for their PGY2 training program. Fifteen of the 16 PGY1 participants (94%) selected surgery as their specialized training track for PGY2.

## Discussion

Our study, involving 44 medical students from Taiwan, evaluated the impact of a custom-designed laparoscopic training workshop on surgical career aspirations and skill acquisition. The primary outcome revealed a post-workshop increased interest in surgery, with the mean score rising from 8.15 to 9.3. Additionally, participants demonstrated a significant reduction in time required for laparoscopic tasks, underscoring the workshop’s effectiveness. A post-training sentiment analysis further highlighted a strong inclination toward surgery among most participants. Collectively, these findings emphasize the workshop’s dual impact on enhancing technical skills and shaping career aspirations.

The findings of our study underscore the profound influence of laparoscopic workshops on medical students’ perspectives regarding a surgical career. Notably, there was a marked increase in post-workshop scores, indicating heightened interest in pursuing surgery as a potential career path. This upward shift aligns with the notion that hands-on workshops, such as the one we conducted, offer more than just skill acquisition [10, 14–16]; they also show a tangible and immersive experience that can significantly shape career aspirations [17, 18]. This is particularly evident when considering the significant reduction in time taken by participants to complete laparoscopic tasks post-training, a testament to the workshop’s effectiveness at enhancing technical proficiency. Our research findings resonate with the outcomes presented by Bonrath et al., emphasizing the potential of laparoscopy simulations in skill acquisition for medical students [19].

Delving into the reasons behind our findings, several factors have emerged as potential contributors. Practical exposure through these workshops demystifies the realm of surgery, making it more accessible and less daunting for students. This hands-on approach not only augments the often-limited surgical exposure in standard medical curricula [20] but also could address and rectify any misconceptions students may harbor about the specialty. Furthermore, the sense of accomplishment derived from mastering intricate laparoscopic techniques can ignite a deeper passion for the field [21], bolstered by the increased confidence in handling surgical instruments and understanding the immediate impact of surgical interventions [22–24]. During these workshops, students gain practical skills and benefit from interactions with seasoned surgeons and their peers. This combination of hands-on training and collaborative dialogue offers a comprehensive learning environment that can notably shape students’ career decisions [25, 26].

In comparison to other specialties, the hands-on nature of surgery, as experienced through these workshops, offers students a unique point of reference. Sedaghat et al. [27] underscore the significance of hands-on experience influencing career determinations. Their study revealed that students attributed hands-on experience as pivotal in defining their career aspirations or cultivating a newfound interest in the surgical field. This observation is corroborated by O’Herrin et al. [28], who observed a marked escalation in the inclination for surgery as a profession, rising from 7% prior to clerkship to 40% following an intervention. While other interventions may influence career choices by focusing on theoretical knowledge or observational experiences, the tangible skills and insights from our laparoscopic workshop facilitate a more comprehensive understanding. This hands-on experience, coupled with the opportunity for mentorship and the validation of pre-existing interests in surgery, positions our workshop as a pivotal intervention in shaping the future aspirations of budding medical professionals.

Evaluation of the outcomes of our training workshops underscored the effectiveness of this intervention, particularly evidenced by the significant reduction in time required by participants to complete the tasks post-training. When benchmarked against commercialized training workshops that focus on similar tasks, the efficacy of our workshop was comparable. This suggests that our tailored approach to training is beneficial and stands on par with established commercial training paradigms regarding advancing students’ proficiency in these tasks [29].

Incorporating laparoscopic skill training into medical education is becoming increasingly pertinent. While it may seem redundant given the limited procedural responsibilities of students, its importance is magnified by the rising prominence of minimally invasive surgery [30]. Brief yet immersive interactions with laparoscopic techniques can profoundly influence students [31, 32], bolstering their confidence and fostering more engaged participation during clerkships [33]. This foundational experience paves the way for their potential hands-on roles in laparoscopic surgeries in the future.

## Conclusions

Our prospective study evaluated a custom-designed laparoscopic training workshop for medical students in Taiwan. Our results revealed a significant enhancement in technical proficiency, as evidenced by reduced post-training task completion times. Moreover, the workshop markedly influenced students’ career aspirations toward surgery, with post-workshop scores indicating a heightened interest. The efficacy of our workshop was comparable to established training paradigms, emphasizing its potential role in medical curricula. The hands-on experience provided by such workshops not only bolsters skill acquisition but can also play a pivotal role in shaping the career trajectories of emerging medical professionals.

## Data Availability

The data supporting the findings of this study are available from the corresponding author upon reasonable request. To obtain the data, please contact Dr. Chih-I Chen at jimmyee0901@gmail.com.

## Abbreviations

PGY: postgraduate year
